# Island biogeography theory explains the genetic diversity of a fragmented rock ptarmigan (*Lagopus muta*) population

**DOI:** 10.1002/ece3.5007

**Published:** 2019-02-27

**Authors:** Jean‐Marc Costanzi, Øyvind Steifetten

**Affiliations:** ^1^ Department of Natural Sciences and Environmental Health University of South‐Eastern Norway Bø i Telemark Norway

**Keywords:** alpine birds, habitat isolation, landscape genetics, microsatellites

## Abstract

The island biogeography theory is one of the major theories in ecology, and its applicability to natural systems is well documented. The core model of the theory, the equilibrium model of island biogeography, predicts that species diversity on an island is positively related to the size of the island, but negatively related by the island's distance to the mainland. In recent years, ecologists have begun to apply this model when investigating genetic diversity, arguing that genetic and species diversity might be influenced by similar ecological processes. However, most studies have focused on oceanic islands, but knowledge on how the theory applies to islands located on the mainland (e.g., mountain islands, forest islands) is scarce. In this study, we examined how the size and degree of isolation of mountain islands would affect the genetic diversity of an alpine bird, the rock ptarmigan (*Lagopus muta*). Within our study area, we defined the largest contiguous mountain area as the mainland, while smaller mountains surrounding the mainland were defined as islands. We found that the *observed heterozygosity *(*H*_o_) was significantly higher, and the *inbreeding coefficient* (*F*_is_) significantly lower, on the mainland compared to islands. There was a positive significant relationship between the *unbiased expected heterozygosity *(*H*_n.b._) and island size (log km^2^), but a negative significant relationship between *H*_o_ and the cost distance to the mainland. Our results are consistent with the equilibrium model of island biogeography and show that the model is well suited for investigating genetic diversity among islands, but also on islands located on the mainland.

## 1 INTRODUCTION

Islands have for decades been a model of choice for ecologists, and because they are closed environments, islands are well suited for ecological studies (MacArthur & Wilson, 1967). The island biogeography theory developed by MacArthur and Wilson (1967) laid the foundation for modern biogeography and provided important contributions to ecology, evolution, and conservation biology (Losos & Ricklefs, 2009). The core model of the theory, the equilibrium model of island biogeography, predicts that the number of species on an island is dependent on two factors: the extinction rate and the immigration rate (Whittaker & Fernandez‐Palacios, 2007). These two factors act in balance and are influenced, respectively, by two different geographical characteristics of the island: area and distance to the mainland. The model predicts a negative relationship between species persistence and island area (as a proxy of the carrying capacity of the island), and between distance to the mainland and immigration rate. This means that larger islands and islands closer to the mainland are predicted to have a lower extinction rate and a higher immigration rate, respectively, than smaller and more isolated ones, both resulting in higher species diversity. The importance of both parameters has been verified by several empirical studies (Kalmar & Currie, 2006; Power, 1972; Simpson, 1974).

Recently, using predictions from the equilibrium model of island biogeography, ecologists have investigated genetic diversity among islands on the assumption that it may have many similarities with species diversity, and that it might be influenced by similar processes (Losos & Ricklefs, 2009). Vellend and Geber (2005) argued that random extinctions of species in island communities could be similar to the loss of alleles due to genetic drift, and that immigration could counteract the loss of genes by bringing new alleles in a population or counteract the loss of biodiversity by bringing new species into a community. They also found that genetic diversity and species diversity on islands were strongly correlated, suggesting a link between the two parameters (Vellend, 2003; Vellend & Geber, 2005). Several studies have for a number of different taxa found that genetic diversity was lower on islands compared to the mainland (Băncilă & Arntzen, 2016; Frankham, 1997; Mcglaughlin et al., 2014; Wang et al., 2014), and that it can be related to some of the predictions of the equilibrium model of island biogeography (Francisco, Santiago, Mizusawa, Oldroyd, & Arias, 2016; García‐Verdugo et al., 2015; Hurston et al., 2009; Mcglaughlin et al., 2014; Sato et al., 2017; Wang et al., 2014; Yamada & Maki, 2012). Furthermore, a significant relationship between genetic diversity and island area (Frankham, 1996; Hill, Loxterman, & Aho, 2017; Sato et al., 2017; Wang et al., 2014), and between genetic diversity and distance to the mainland (Francisco et al., 2016; García‐Verdugo et al., 2015; Yamada & Maki, 2012) has also been found. While several studies have examined the relationship between island area, distance to the mainland and genetic diversity, to our knowledge only Hill et al. (2017) and Mcglaughlin et al. (2014) explicitly tested for the equilibrium model of island biogeography.

Of the many studies investigating the relationship between genetic diversity and the different parameters of the island biogeography theory, few have been conducted on population living on the continent (e.g., mountain islands, forest islands or lakes; Hänfling & Brandl, 1998; Hänfling, Hellemans, Volckaert, & Carvalho, 2002), and to our knowledge only two have been conducted on mountain islands (Epps, Palsboll, Wehausen, Roderick, & McCullough, 2006; Hill et al., 2017). For continental populations, an island can be described as a patch of suitable habitat surrounded by unsuitable matrix habitat (Haila, 2002). For this study, we used the Scandinavian rock ptarmigan (*Lagopus muta muta*) as a model species, a bird that inhabits large and small mountain fragments within the study area, to investigate a possible relationship between genetic diversity and the island biogeography theory. We chose islands of various size and distance to the mainland (i.e., a disproportionally large and contiguous mountain area) to see whether there was (a) a difference in genetic diversity between the mainland and the mountain islands, (b) a relationship between genetic diversity and the size of an island, and (c) a relationship between genetic diversity and the distance to the mainland. We expected that genetic diversity would be higher on the mainland compared to the islands, and that genetic diversity would be higher on larger islands and on islands closer to the mainland.

## 2 MATERIALS AND METHODS

### 2.1 Study species and study area

The rock ptarmigan is a distinctive montane bird living above the tree line distributed throughout the northern hemisphere (Watson & Moss, 2008). Globally it is listed as Least Concern due to its extensive distribution and local large population size (Birdlife International, 2015), but the species is steadily declining in Scandinavia and in Southern Europe (Birdlife International, 2015; Lehikoinen, Green, Husby, Kålås, & Lindström, 2014). The rock ptarmigan is an ample flyer and dispersal distances up to 300 km have been reported (Gardarsson & Bossert, 1997), but it tends to avoid crossing unsuitable matrix habitat (Bech et al., 2013; Bech, Boissier, Drovetski, & Novoa, 2009; Novoa et al., 2014).

**Figure 1:**
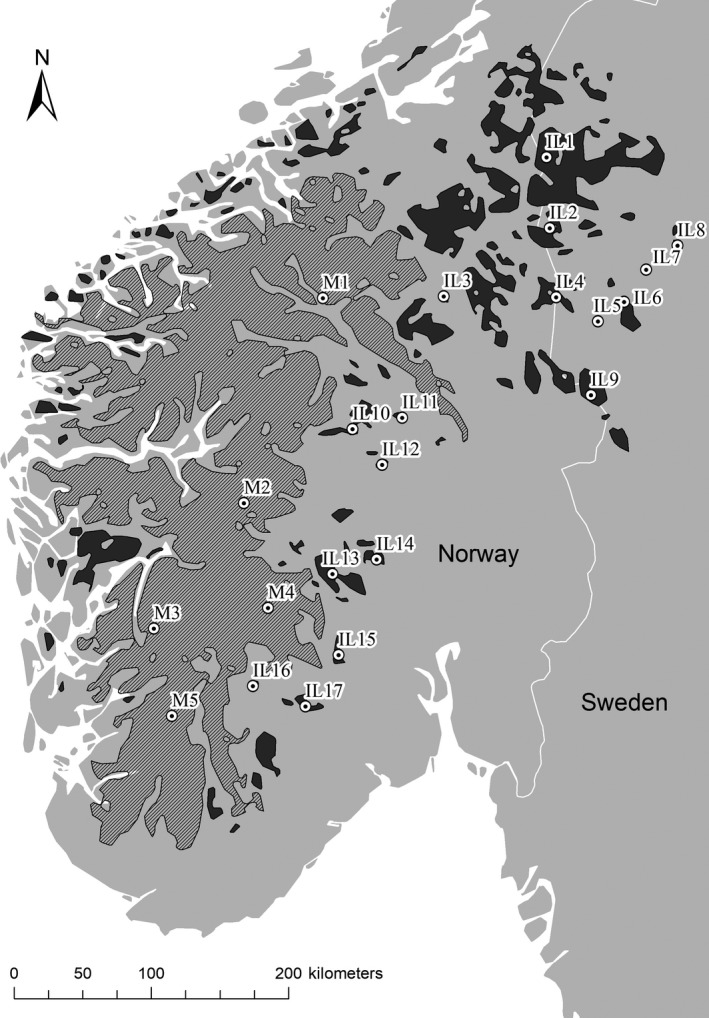
Map of the study area showing the 22 sampling sites (dots) and their respective ID numbers. The hatched area is defined as the “mainland” and the dark gray areas as the “islands”

The study was conducted within the Fennoscandian mountain range situated in southern Norway and into central Sweden. The study area extends approximatively 550 km in latitude and 450 km in longitude. Within the frame of the model of island biogeography, the study area presented a great advantage as it contained a large contiguous mountain area that could represent the mainland, and several surrounding mountain fragments that could represent islands. We defined all areas above the tree line as potential habitat for the rock ptarmigan, and these areas were mapped using interpolation of 357 point observations of the tree line altitudes in Norway and Sweden (see Kullman, 1979, Moen, 1998). A map of the tree line was created using a TIN model with ArcMap 10.3 (ESRI, 2015a). Mountain fragments with a size less than 0.5 km^2^ were removed from the map altogether as they were considered too small to be important as rock ptarmigan habitat. Because daily dispersal distances of grouse are often more than 2.5 km (Caizergues & Ellison, 2002; Hörnell‐Willebrand, Willebrand, & Smith, 2014), areas separated by less than 2.5 km were connected and treated as one single mountain unit. The consolidation of mountain areas into mountain units was performed to reduce the initial number of 5,900 small and large fragments, and to highlight the mainland from the surrounding islands (Figure 1).

### 2.2 Data collection

For analyses, we used two types of genetic material: fecal pellets collected on the ground, and feathers collected on the ground and from shot birds. Samples were collected from 22 different sites: five situated on the mainland and 17 on the surrounding islands (Figure 1, Table 1). Collection of fecal pellets and feathers on the ground was performed during February‐April in the years 2013–2016 (but the years 2013 and 2014 for site IL17 only, and samples from site M2 were collected during fall 2016). Samples were randomly collected within already designated areas selected for their geographical characteristics, like degree of isolation (i.e., distance from the mainland) and area size. Each site was only sampled for a maximum of 2 days. To avoid collecting samples from the same individual, a distance of 500 m was set between sampling sites, with the exception of when several birds were observed together or when several snow‐roosts was discovered. Samples from shot birds were collected during the hunting seasons of 2012, 2014, 2015, and 2016 (Appendix S1).

**Table 1:** Genetic indices and geographical characteristics for each sampling unit

Site ID	Landscape type	Area (km^2^)	Euclidian distance	Cost distance	*N*	Average elevation	Ar.	*H*_o_	*H*_e_	*H*_n.b._	*F*_is_
IL1	Island	2,492	89	20,332	92	1,032	2.56	0.61	0.66	0.67	0.09
IL2	Island	290	82	24,194	24	1,057	2.47	0.56	0.63	0.64	0.13
IL3	Island	25	16	15,524	37	1,238	2.45	0.57	0.62	0.63	0.10
IL4	Island	216	80	33,653	16	1,056	2.46	0.50	0.63	0.67	0.27
IL5	Island	10	115	58,993	17	1,061	2.42	0.49	0.60	0.62	0.22
IL6	Island	120	131	73,598	22	924	2.53	0.55	0.64	0.66	0.17
IL7	Island	14	157	72,077	31	1,113	2.47	0.55	0.63	0.64	0.15
IL8	Island	15	183	71,269	19	940	2.16	0.45	0.53	0.53	0.15
IL9	Island	294	87	48,983	20	927	2.30	0.53	0.57	0.59	0.09
IL10	Island	113	4	4,661	24	1,273	2.46	0.66	0.62	0.63	−0.03
IL11	Island	15	20	20,128	17	1,137	2.32	0.53	0.57	0.59	0.10
IL12	Island	28	44	29,432	24	1,220	2.28	0.45	0.57	0.58	0.24
IL13	Island	289	3	2,971	48	1,134	2.52	0.55	0.65	0.66	0.16
IL14	Island	83	39	21,677	32	1,134	2.38	0.54	0.60	0.61	0.11
IL15	Island	70	11	9,247	28	1,068	2.47	0.56	0.63	0.64	0.12
IL16	Island	23	3	3,494	15	1,126	2.49	0.61	0.62	0.65	0.07
IL17	Island	76	18	16,945	25	1,127	2.38	0.52	0.60	0.62	0.15
M1	Mainland	42,702	NA	NA	31	NA	2.53	0.65	0.65	0.66	0.03
M2	Mainland	42,702	NA	NA	16	NA	2.44	0.64	0.61	0.63	0.00
M3	Mainland	42,702	NA	NA	20	NA	2.44	0.58	0.62	0.63	0.08
M4	Mainland	42,702	NA	NA	26	NA	2.49	0.56	0.63	0.64	0.13
M5	Mainland	42,702	NA	NA	32	NA	2.50	0.64	0.64	0.65	0.01

Notes

Number of samples (*N*), allelic richness (Ar.), observed heterozygosity (*H*_o_), expected heterozygosity (*H*_e_), unbiased expected heterozygosity (*H*_n.b._), inbreeding coefficient (*F*_is_). The Euclidian distance is presented as kilometers to the mainland, the cost distance as the minimum cumulated distance below the tree line to the mainland (in meters) and average elevation as meters above sea level.

### 2.3 DNA analysis

Depending on the type of genetic sample, two different protocols of DNA extraction were used. The DNA from feathers was extracted with the “DNeasy Blood & Tissue kit” (Qiagen, Cat. No. 69506), while DNA from fecal pellets was extracted with the “QIAamp DNA Stool Mini Kit” (Qiagen, Cat. No. 51504). To maximize the final yield of extracted DNA, the “DNeasy Blood & Tissue” procedure was modified with the addition of 5 µl of DTT 1 M to the ATL buffer during the first step of the protocol. We also modified the “QIAamp DNA Stool Mini Kit” protocol, where we adjusted the quantity of buffer ASL from 1.4 to 1.6 ml. Because fecal pellets of the rock ptarmigan are impossible to differentiate from fecal pellets of the willow grouse (*L. lagopus*), every sample was amplified with the species‐specific mtDNA primers Lagsp3F, Lag3R, and Mut3R (see Nyström et al., 2006). PCR conditions followed Nyström et al. (2006), and the results were read via polyacrylamide gel electrophoresis (see Bergan, Sæbø, & Parker, 2016). Rock ptarmigans were identified by an amplicon size of 212 bp, and willow grouse by an amplicon size of 154 bp.

For this study, we used 14 of 16 microsatellite markers previously developed for the rock ptarmigan (see Costanzi, Bergan, Saebo, Jenkins, & Steifetten, 2018), the reason being that two of the markers (Mut12 and Mut20) had a high frequency of null alleles. PCR amplification was performed with forward primers labeled with FAM, NED, PET, or VIC fluorescent dyes. For the PCR reaction, we used 6 µl of “Qiagen Taq PCR mastermix” (Qiagen, Cat. No. 201445; for a final concentration of 1×), 0.25 µl (20 µM) of each primer, 2 µl of DNA template, and completed the mix with ultrapure water for a total reaction volume of 12 µl. The reaction was realized in a thermal cycler Eppendorf Mastercycler^®^ gradient: 15 min at 95°C, 40 cycles consisting of 30 s at 94°C, 1 min 30 s at 60°C, 1 min at 72°C, and finally 10 min at 72°C. To reduce the time and cost of the analyses, microsatellite markers were multiplexed in five different reactions. A negative control was included in every reaction and analyzed in parallel with the samples. If a positive signal was detected, the results were discarded and the entire run was repeated. Genotyping was performed on a 3130xl Genetic analyser (Applied Biosystems) with 1.5 µl of PCR product added in a mix containing 9.7 µl of formamide (Thermo Scientific™, Cat. No. 17899) and 0.3 µl of GeneScan 500LIZ dye Size Standard (Applied Biosystems™, Cat. No. 4322682). Allele scoring was performed using Genemapper software V5.0 (Applied Biosystem) and visually controlled.

To assess the quality of the genotyping procedure, 20% of the samples were run a second time (PCR and genotyping) and a genotyping error rate was calculated. Individuals with more than 10 missing loci were removed from analysis. The multitube approach, which is generally used on noninvasive samples (Miquel et al., 2006), was in our case not considered necessary due to the type and the condition of our samples. DNA from fecal pellets collected during winter has been shown to remain stable, and with high quality, for several days after deposition (Bergan et al., 2016). Microsatellite loci were tested for the presence of null alleles with the software microchecker (Van Oosterhout, Hutchinson, Wills, & Shipley, 2004). Hardy–Weinberg equilibrium and linkage disequilibrium was calculated with the software FSTAT v2.9.3.2 (Goudet, 1995) by performing 6160 randomizations and 9100 permutations, respectively. The level of significance was adjusted with Bonferroni's correction. Sites with less than 15 genotyped individuals were removed from analyses to increase the accuracy of measures on genetic diversity. This number was chosen as it still allows relatively precise results without removing too much of the data (Hale, Burg, & Steeves, 2012). Individual identity analysis was carried out with the software Cervus V3.0.7 (Kalinowski, Taper, & Marshall, 2007), and the minimum number of matching loci was set to 7, and one fuzzy matching was allowed. To test for relatedness (i.e., full siblings) among individuals, we used the software ML‐Relate (Kalinowski, Wagner, & Taper, 2006). We then checked for differences in allele frequencies between datasets with or without full siblings. The comparison was only performed for sampling sites which had more than 5% full siblings over all pairwise comparisons.

Possible introgression with the willow grouse was tested using the software STRUCTURE (Pritchard, Stephens, & Donnelly, 2000), and for the analysis, 45 willow grouse and 30 rock ptarmigan from the same two locations were included. In total, we used ten microsatellite loci common for the rock ptarmigan and the willow grouse: Mut1, Mut2, Mut4, Mut8, Mut14, Mut16, Mut17, Mut18, Mut22, and Mut23 (see Costanzi et al., 2018). Run conditions were the same as described by (Quintela, Thulin, & Höglund, 2010), but every run was repeated ten times instead of five to increase the precision of the results. The average results over ten runs were calculated with CLUMPP version 1.1.2 (Jakobsson & Rosenberg, 2007), and individuals with an average membership coefficient <0.9 were considered hybrids.

### 2.4 Isolation metrics

To measure the distance between the mainland and the different islands, we used the Euclidian distance and cost distance. The Euclidian distance was measured as the shortest straight‐line distance between the mainland and an island, while cost distance was measured using the function “cost distance” in the Spatial Analyst Tools in ArcMap 10.4 (ESRI, 2015b). We used cost distance to complement the Euclidian distance as it tends to perform better and includes stepping stones (Weigelt & Kreft, 2013). It defines the path that will be least costly for an animal depending on habitat type. We assigned a cost of 0 unit per cell (with 50 m cells) for areas above the tree line (i.e., suitable habitat), and a cost of 1 unit per cell for areas below the tree line (i.e., unsuitable matrix habitat). Each cell value was automatically multiplied by the cell resolution in order to account for diagonal movements. The path with the lowest cost was then measured. Island size was measured as the total area (km^2^) above the tree line.

### 2.5 Genetic diversity analyses

We measured genetic diversity using four different indices: observed heterozygosity (*H*_o_), expected heterozygosity (*H*_e_), unbiased expected heterozygosity (*H*_n.b._), and allelic richness (Ar.). We also calculated the inbreeding coefficient *F*_is_ as a supplement to the four indices. *H*_o_, *H*_e_, *H*_n.b._, and *F*_is_ were calculated with the software Genetix V4.05 (Belkhir, Borsa, Chikhi, Raufaste, & Bonhomme, 2004), while Ar. was calculated with rarefaction method using ADZE 1.0 (Szpiech, Jakobsson, & Rosenberg, 2008) for four individuals (i.e., the minimum number of individuals without missing data for all loci and sampling sites). The genetic indices were selected in order to observe different ecological processes; *H*_e_, *H*_n.b._, and Ar. are mainly driven by population size (Palstra & Ruzzante, 2008; Petit, Mousadik, & Pons, 1998), while *H*_o_ and *F*_is_ are more influenced by inbreeding (Keller & Waller, 2002). We tested for the effect of missing data on loci Mut17 and Mut24 by performing a correlation test between *H*_o_, *H*_n.b._, Ar., and *F*_is_, calculated with 14 and 12 loci, respectively.

### 2.6 Statistical analyses

We used a Wilcoxon signed‐rank test to see whether there were any differences in genetic diversity and *F*_is_ between the mainland and the islands. Collinearity among independent variables was checked with variance inflation factors, and only variables with scores <3 were selected (Zuur, Ieno, & Elphick, 2010). We used linear models calculated with the software R v3.3.2 (R Core Team, 2016) to check for relationships between the independent variables (cost distance/Euclidian distance and island area) and the dependent variables (*H*_e_, *H*_n.b._, *H*_o_, Ar., and *F*_is_). For each index, six candidate models were created with the independent variables: log(area), log(cost distance), log(Euclidian distance), log(area) + log(cost distance), log(area)  + log(Euclidian distance), and finally, an intercept model using 1 as an independent variable. A model selection based on Akaike's information criterion corrected for sample size (AIC_c_; Burnham, Anderson, & Huyvaert, 2011) was used with the function model.sel from the R package MuMIn (Barton, 2016). Models with the lowest AIC_c_ score were selected. If two models had a delta AIC_c_ (ΔAIC_c_) lower than 2, only the most parsimonious model was selected to avoid the “pretending variable” issue (Anderson, 2008). Indices with zero in their 95% confidence intervals were considered noninformative (Arnold, 2010).

The effect of sampling effort on genetic indices was examined by comparing the total area sampled for each site with *H*_n.b_, *H*_o_, Ar., and *F*_is_. To estimate the total area sampled, we created a Minimum Convex Polygon around each collected sample per site using ArcMap 10.4 (ESRI, 2015b). We treated the area of each polygon as a proxy for sampling effort, and linear models were used to test for relationships between the area sampled and genetic diversity. To analyze the amount of genetic connectivity between islands (island–island), between island and the mainland (island–mainland), as well as between different sites on the mainland (mainland–mainland), we used pairwise *F*_st_ calculated with the software Arlequin (Ver 3.5; Excoffier & Lischer, 2010). We then used a linear regression to see whether the pairwise *F*_st_ of mainland–mainland was significantly different from the pairwise *F*_st_ of island–mainland and island–island. The effect of sampling site elevation on the genetic indices *H*_e_, *H*_n.b_, Ar., and *F*_is_ was analyzed with linear regression models.

## 3 RESULTS

In total, 1,042 genetic samples were collected; 804 were fecal pellets and feathers collected on the ground, and 238 were feathers received from hunters. The mainland constituted an area of 42,702 km^2^, while islands ranged in size from 10 km^2^ (IL5) to 2,492 km^2^ (IL1; Table 1 and Figure 1). The Euclidian distance and the cost distance from the islands to the mainland varied from 3 km (IL13 and IL16) to 183 km (IL8) and from 2,971 (IL13) to 73,598 (IL6), respectively (Table 1).

### 3.1 Data assessment

After removing samples from the willow grouse, poor quality samples, and duplicates (66 samples), 616 individual rock ptarmigan samples were available for analyses. Of these, 491 came from islands and 125 came from the mainland. We found no Hardy–Weinberg disequilibrium or linkage disequilibrium after Bonferroni's correction (respectively, *p* < 0.00016 and *p* < 0.000549). Average microsatellite error rate over all loci was 2.6% (range: 0.76%–7.4%). The total dataset had less than 5% of missing data. For IL4 data were missing in 14 and 11 individuals (of 16) for Mut17 and Mut24, respectively, and for IL5 in 15 individuals (of 17) for Mut24. The genetic indices *H*_o_, *H*_n.b_, Ar., and *F*_is_ were found to be highly correlated when calculated with 12 or 14 loci (Pearson's correlation: 0.96, 0.92, 0.92, and 0.97, respectively), and we therefore decided to use 14 loci for the rest of the analysis. We found no significant relationship between *H*_o_, *H*_n.b_, Ar., *F*_is_ and sampling effort (linear regressions, *p* > 0.2), or between *H*_o_, *H*_n.b_, Ar., *F*_is_, and the average altitude per site (linear regressions, *p* > 0.1).

No hybrids were detected among the 30 rock ptarmigan and 45 willow grouse that were tested. The average membership coefficient for all runs was 0.992 for the rock ptarmigan (range: 0.934–0.998) and 0.993 for the willow grouse (range: 0.980–0.998). When analyzing relatedness among individuals, we found that overall sites 1.99% comparisons showed potential full siblings (range: 0.83% for site M2%–14.71% for site IL11). Sites with more than 5% full siblings (sites IL8, 9, 11, and 12) did not significantly affect allele frequencies (Wilcoxon signed‐rank test, *p* ≥ 0.30), and we therefore decided to keep these individuals for further analyses (Waples & Anderson, 2017).

Because we found a significant relationship between expected heterozygosity (*H*_e_) and the number of samples per site (linear regression, *p* < 0.05), we replaced *H*_e_ by the unbiased expected heterozygosity (*H*_n.b._) to compensate for differences in sample size (Petit et al., 1998). No significant relationship was, however, found between the number of samples per site and *H*_o_, *H*_n.b_, Ar., and *F*_is_ (linear regression, *p* > 0.1).

### 3.2 Genetic diversity

The number of alleles per loci for the 616 samples used in analyses lie between 2 (Mut24) and 23 (Mut17). Average allelic richness (Ar.) per site over all loci ranged from 2.16 (IL8) to 2.56 (IL1), unbiased expected heterozygosity (*H*_n.b._) ranged from 0.53 (IL8) to 0.67 (IL1 and IL4), observed heterozygosity (*H*_o_) ranged from 0.45 (IL12 and IL8) to 0.66 (IL10), and the inbreeding coefficient (*F*_is_) ranged from −0.03 (IL10) to 0.27 (IL4; Table 1).

**Figure 2:**
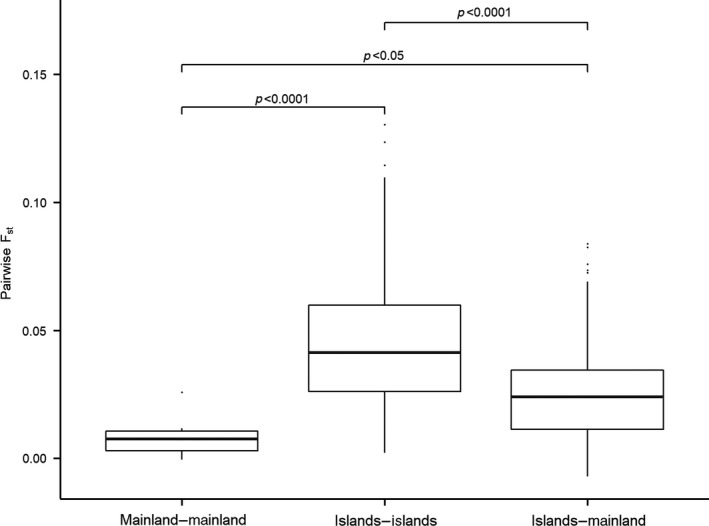
Box plots showing the pairwise *F*_st_ calculated for all sites, and organized into three categories: between sites on the mainland, between islands, and between sites on the mainland and the different islands. Median values, 25th and 75th percentiles, and 95% confidence intervals are shown. The *p*‐values indicate if a significant pairwise *F*_st_ was observed between the different groups

**Figure 3:**
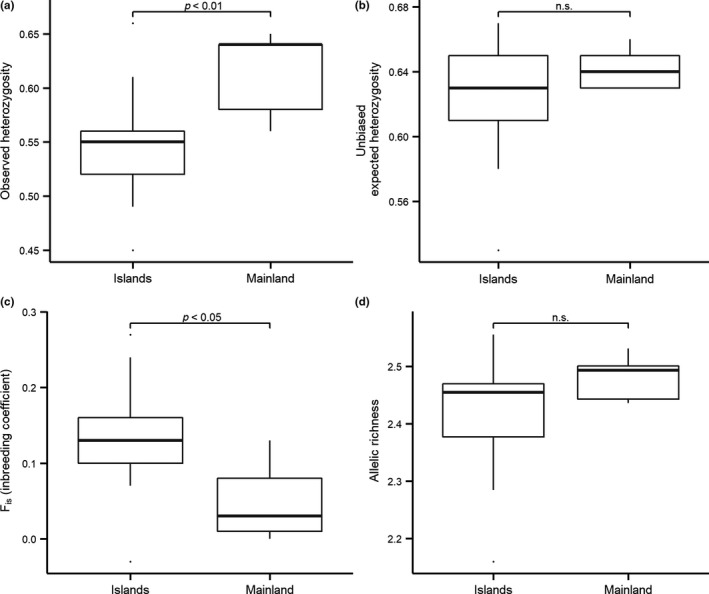
Box plots showing the genetic indices for sites on the mainland and on the islands: (a) observed heterozygosity (*H*_o_), (b) unbiased expected heterozygosity (*H*_n.b._), (c) inbreeding coefficient (*F*_is_), (d) allelic richness (Ar.). Median values, 25th and 75th percentiles, and 95% confidence intervals are shown. The *p*‐values indicate if a significant difference between islands and mainland was observed (*p* < 0.01 or *p* < 0.05) or if the difference was nonsignificant (n.s.)

The genetic distance was low between sites on the mainland (average pairwise *F*_st_: 0.008), and significantly lower (linear regression, *p* < 0.0001) than between islands (average pairwise *F*_st_: 0.041), as well as between islands and the mainland (linear regression, *p* < 0.05; average pairwise *F*_st_: 0.024; Figure 2). For every genetic index tested, the mainland showed a higher genetic diversity than the islands (Figure 3). The difference was highly significant for *H*_o_ (Wilcoxon signed‐rank test, *p* < 0.01; Figure 3a), and for *F*_is_, it was significantly lower on the mainland than on the islands (Wilcoxon signed‐rank test, *p* < 0.05; Figure 3c).

**Figure 4:**
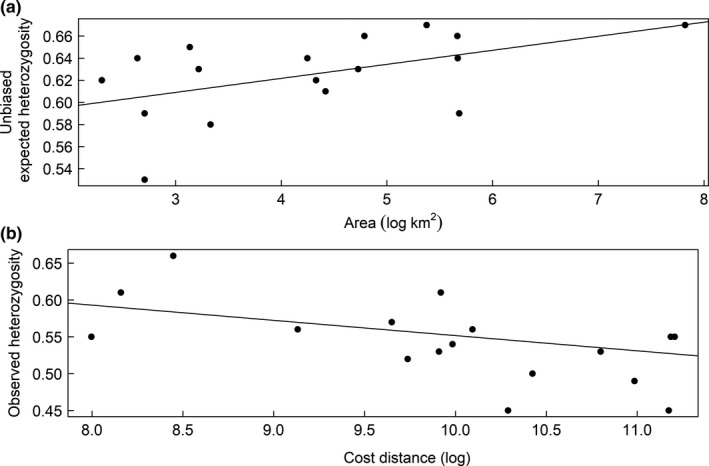
Relationship between (a) unbiased expected heterozygosity (*H*_n.b._) and area (log km^2^; *R* ^2^: 0.21), and (b) observed heterozygosity (*H*_o_) and Euclidian distance (log km; *R* ^2^: 0.33) for the 17 islands

Observed heterozygosity (*H*_o_) was best explained by the cost distance to the mainland, while the unbiased expected heterozygosity (*H*_n.b._) was best explained by area (Table 2). Allelic richness was best explained by area, but the index was considered noninformative (lower and upper confidence interval: −0.016 and 0.315). However, *F*_is_ was best explained by the cost distance and was considered informative (lower and upper confidence interval: 0.0002 and 0.047), but the difference in AIC_c_ score corrected for sample size (ΔAIC_c_) was not significantly different from the intercept model (ΔAIC_c_: 1.74, Table 2). The best models showed that individuals from more isolated islands were more likely to have a low *H*_o_ (adjusted *R* ^2^: 0.33), and that individuals from islands of larger size were more likely to have a high *H*_n.b._ (adjusted *R* ^2^: 0.21; Figure 4 and Table 3).

**Table 2:** The change in AIC_c_ (ΔAIC_c_) from the best model for all four dependent variables: unbiased expected heterozygosity (*H*_n.b._), observed heterozygosity (*H*_o_), inbreeding coefficient (*F*_is_), and allelic richness (Ar.)

Model (log)	Δ AIC_c_			
	*H*_n.b._	*H*_o_	*F*_is_	Ar.
Area	**0**	4.99	4.29	0
Cost distance	2.86	**0**	0	1.27
Distance	4.06	2.32	0.50	2.59
Area + cost distance	1.87	1.28	3.40	1.47
Area + distance	2.04	1.86	3.45	1.81
None	2.17	4.83	1.74	0.96

Note

The selected models are in bold.

**Table 3:** Model results selected by AIC_c_ for the unbiased expected heterozygosity (*H*_n.b._) and observed heterozygosity (*H*_o_), their respective effect size (*β*), standard error (*SE*), lower (LCI), and upper (UCI) 95% confidence interval

Selected model	*β*	*SE*	LCI	UCI
*H*_n.b._ ~ log(area)	0.013	0.006	0.001	0.024
*H*_o_ ~ log(cost distance)	−0.032	0.011	−0.055	−0.009

## 4 DISCUSSION

The main results of this study support the predictions of the equilibrium model of island biogeography, in which genetic diversity was found to be significantly higher on the mainland compared to the surrounding islands. We found a significant relationship between observed heterozygosity (*H*_o_) and distance to the mainland, and between the unbiased expected heterozygosity (*H*_n.b._) and island size. This shows that the theory is well suited for not only analyzing species diversity among oceanic islands, but also genetic diversity within a fragmented mountain landscape. It seems that for isolated populations, the immigration rate is insufficient to compensate for genetic drift, and suggest that the future viability of such populations could be imperiled by extinction vortex processes.

Although the interpretation of genetic data is very much a result of data quality, we believe that the methods and analyses used in this study have created a robust dataset, and that any biases have been taken into consideration. For being noninvasive samples, the genotyping error rate was extremely low (Luikart et al., 2008) and is likely due to the type and time of year when the samples were collected (i.e., fecal pellets with a high fiber content but with a low water content, and during winter on snow with low temperatures; see Bergan et al., 2016). We also selected species‐specific microsatellites loci with a tetra‐nucleotide motif, which are less prone to slippage, to reduce the genotyping error rate (Costanzi et al., 2018). Although the presence of close relatives in some sites could lead to changes in allele frequencies resulting in biased genetic estimates (Goldberg & Waits, 2010), such changes were not found for sites with the highest number of full siblings. Waples and Anderson (2017) also argue that one should be cautious when removing siblings as they are a natural part of any population. We therefore believe that the number of siblings in our dataset is insufficient to create a strong bias, and removing them would actually decrease the precision of our genetic indices (Waples & Anderson, 2017). Another parameter that has been shown to affect genetic diversity in a mountain landscape, and which possibly could bias the results, is the average elevation but no such effect was found in our study area. Thus, it was not included in the model selection. One could also argue whether or not our study area could be treated as a mainland/island system, but pairwise *F*_st_ tests confirmed that islands are significantly more isolated than sites situated on the mainland, thus supporting this division. The *F*_st_ values observed between islands and the mainland, and between islands indicate that there is low connectivity between these sites, in addition to stronger genetic drift on islands compared to the mainland due to small population size (Allendorf, Luikart, & Aitken, 2013). On the other hand, the lower *F*_st_ values observed on the mainland is likely to be a direct consequence of larger population size and a higher connectivity between subpopulations (Rannala & Hartigant, 1995).

### 4.1 Comparison of the genetic indices between islands and the mainland

Genetic diversity was consistently higher on the mainland compared to the islands for every genetic diversity index measured (*H*_n.b._, Ar., *H*_o_), while the opposite was true for the level of inbreeding (*F*_is_). These results are in line with the meta‐analysis carried out by Frankham (1997) on different animal and vegetal taxa, who found that in 48 of 57 studies heterozygosity (observed or expected) was higher on the mainland than on islands. Similar results have also been found in several other studies showing that the mainland exhibits a higher genetic diversity than islands (Băncilă & Arntzen, 2016; García‐Verdugo et al., 2009; Mason, Browning, & Eldridge, 2011; Mcglaughlin et al., 2014; Wang et al., 2014; White & Searle, 2008; Yamada & Maki, 2012). A lower genetic diversity on islands has also been described by Wright (1940) in his island model, which predicts that islands with large populations and regular occurring gene flow between the island and the mainland will have the same genetic diversity as the mainland. However, islands with small populations and reduced gene flow are expected to experience increased genetic drift resulting in a change of allele frequency (Hedrick, 2010). Although gene flow to some extent can counteract the effect of genetic drift, genetic diversity is still likely to decrease if it is too low (Hedrick, 2010). A genetic study of the rock ptarmigan in Western Europe found that the Pyrenean population had a lower genetic diversity than populations throughout the Scandinavian mountain range and was explained by past and present isolation of the Pyrenean population, in addition to its small population size (Caizergues, Bernard‐Laurent, Brenot, Ellison, & Rasplus, 2003).

Our study showed that only *H*_o_ and *F*_is_ were significantly different between islands and the mainland. One explanation could be that the different genetic indices are not driven by the same ecological processes and do not explain the same mechanisms. Depending on landscape characteristics and the ecological requirements of the species, the significance of each index may vary. For example, *H*_o_ and *F*_is_ are more likely to be influenced by inbreeding (Keller & Waller, 2002), meaning that these indices are more strongly affected by immigration rate (Allendorf et al., 2013). Our results could therefore indicate that there is reduced gene flow between islands and the mainland. Unsuitable matrix habitat (i.e., all area below the tree line) seems to act as a barrier for movement, restricting immigration of new individuals, and hence new alleles into the population. Although the rock ptarmigan is an ample flyer, it tends to avoid dispersing over unsuitable matrix habitat, and it has been shown to follow patches of more suitable habitat during dispersal (Novoa et al., 2014). This was supported by Bech et al. (2009) who showed that 18 km of unsuitable habitat in the Pyrenees was enough to isolate and to genetically differentiate rock ptarmigan populations.

While *H*_o_ and *F*_is_ could be explained by a lack of connectivity, the nonsignificant results for *H*_n.b._ and Ar. might be due to island size. These two indices are mostly influenced by population size (Nei, Maruyama, & Chakraborty, 1975; Palstra & Ruzzante, 2008; Petit et al., 1998), and in many cases, population size can be correlated with area size (Frankham, 1996). Thus, some of the large islands within our study area (e.g., IL1, IL2, IL4, and IL13) might have populations large enough to obtain values of *H*_n.b._ and Ar. similar to the mainland, and they are probably the reason why we do not find a difference between islands and the mainland for these two indices.

### 4.2 Relationship between island area and the genetic indices

A relationship between genetic diversity and island area has been documented in several studies (Hänfling et al., 2002; Knaepkens, Bervoets, Verheyen, & Eens, 2004; Sato et al., 2017; Wang et al., 2014; White & Searle, 2007), but to our knowledge, only two have been conducted in montane landscapes (Epps et al., 2006; Hill et al., 2017). Our results showed that *H*_n.b._ was best explained by area size, and this is in concord with previous findings for different types of landscapes and taxa (Cheylan, Granjon, & Britton‐Davidian, 1998; Frankham, 1996; Hänfling & Brandl, 1998; Hänfling et al., 2002; Knaepkens et al., 2004; Sato et al., 2017; Wang et al., 2014; White & Searle, 2007). When comparing large and small populations, the general rule is that small populations have fewer alleles and are more strongly affected by random genetic drift than large populations. If small populations are also isolated, genetic drift may be enhanced (Allendorf et al., 2013), and this is probably true for several of the small populations in our study area. Although area size in some cases might be a poor predictor of population size, as it can be influenced by habitat availability and population density (Wang et al., 2014), we believe that our delineation of potential rock ptarmigan habitat is a good approximation of population size, and we therefore expect the two parameters to be correlated. We also suggest that a bottleneck could be the reason for the decrease in *H*_n.b._. However, we only found one site (IL4) that might have had a potential bottleneck in the past.

Contrary to what was expected we did not find a significant relationship between Ar. and area size (e.g., results from Hill et al., 2017), and one possible explanation could be that population size relative to available habitat differed between sites. The low altitude of some of the sites might have led to overlap in habitat use between the rock ptarmigan and the willow grouse, which in turn could initiate interspecific competition between the two species. Because the willow grouse is likely to outcompete the rock ptarmigan, the end result would be fewer rock ptarmigans relative to available habitat (i.e., measure of potential rock ptarmigan habitat). The observation that some populations have fewer individuals than expected based on available habitat is something that should be considered when interpreting data on genetic diversity.

### 4.3 Relationship between distance to the mainland and the genetic indices

Several studies have found a significant relationship between distance to the mainland and genetic diversity (Francisco et al., 2016; García‐Verdugo et al., 2015; Hill et al., 2017; Yamada & Maki, 2012). Similarly, we found a significant relationship between *H*_o_ and distance to the mainland. This makes sense since *H*_o_ is more influenced by changes in genotype frequencies than in allele frequencies (Keller & Waller, 2002), and that isolation has a stronger effect on genotype frequencies than allele frequencies (Allendorf et al., 2013). Although both the Euclidian distance and the cost distance were highly significant as model parameters, the model including cost distance performed the best. This implies that the rock ptarmigan could be using stepping stones when moving across the landscape, and that cost distance in this study could function as a proxy for isolation. It seems, however, that even though stepping stones to some extent can reduce the effect of isolation, movement over unsuitable matrix habitat is still too low to compensate for the effect of isolation. The low *H*_o_ observed among the severely isolated islands is probably the result of a higher genetic drift than gene flow, resulting in the loss of genetic diversity (Allendorf et al., 2013). There are also studies with no clear relationship between distance to the mainland and genetic diversity (Băncilă & Arntzen, 2016; Hurston et al., 2009; Mcglaughlin et al., 2014; Sato et al., 2017; Soulé & Yang, 1973; Wang et al., 2014). Although there may be different reasons for this, one should keep in mind that isolation can also be affected by the dispersal capacity of the species under study (Frankham, 1997) and to the presence of surrounding islands and their size (Weigelt & Kreft, 2013), making it a complex system to investigate. This complexity might also be the reason why we could not find a significant relationship between the distance to the mainland and *F*_is_, although a positive trend was observed.

## 5 CONCLUSION

The negative effect of either isolation or area size on genetic diversity has been documented in a large number of studies, but surprisingly few have combined the two landscape parameters or related it to the island biogeography theory. This study is an attempt to increase our knowledge on how genetic diversity is affected within such a system, but also if the theory can be applied to mountain islands rather than oceanic islands. Our results support the island biogeography theory, and they show that even for a highly mobile species such as the rock ptarmigan, isolation appears to be the negative factor in maintaining a high genetic diversity. The rock ptarmigan is probably not exceptional in that regard, and any species living above the tree line within a fragmented system are likely to experience similar consequences of isolation. Alpine species are also particularly vulnerable to climate change, in which an upward shift of the tree line is predicted, and also observed, to occur (Harsch, Hulme, McGlone, & Duncan, 2009; Kullman & Öberg, 2009; Rannow, 2013). This could eventually reduce the amount of available habitat and subsequently increase fragmentation (Chamberlain et al., 2012), enhancing the negative effects of isolation and small population size. The theory and the models developed could therefore be useful in predicting the future loss of genetic diversity as a result of climate change.

## CONFLICT OF INTEREST

None declared.

## AUTHOR CONTRIBUTIONS

Ø.S. designed the study; J.M.C. and Ø.S. collected the samples; J.M.C. conducted the laboratory and data analyses; J.M.C. and Ø.S. wrote the manuscript.

## Data Availability

The microsatellite sequences are available on GenBank with accession numbers: MF425753, MF425754, MF425755, MF425756, MF425757, MF425758, MF425759, MF425761, MF425762, MF425763, MF425764, MF425766, MF425767, and MF425768. Results from genotyping, the TIN model and the isolation metrics used are deposited into https://usn.figshare.com/: 10.23642/usn.c.4389815.
