# Supplementary material for: Island biogeography theory explains the genetic diversity of a fragmented rock ptarmigan (Lagopus muta) population
Source: Ecol Evol. 2019 Feb 27;9(7):3837–49. doi: 10.1002/ece3.5007 (PMC6468070; doi:10.1002/ece3.5007)
Supplement: Supplementary file 1 [file ECE3-9-3837-s001.docx]

Annexe 1:

Detailed results of the type of sample collected and the year(s) of collection per site: faecal pellets collected in the winter (Faecal pellets), feather found on the ground during hunting season (Feathers), feather collected from bird shot by hunter during hunting season (Birds shot), year of collection (if the site was sampled over two years, both years are indicated) (Year) and number of individuals (N).

| Location | Type | Fecal pellets | Feathers | Birds shot | Year | N |
| --- | --- | --- | --- | --- | --- | --- |
| IL1 | Island | 75 | 0 | 17 | 2015/2016 | 92 |
| IL2 | Island | 24 | 0 | 0 | 2015 | 24 |
| IL3 | Island | 37 | 0 | 0 | 2015 | 37 |
| IL4 | Island | 16 | 0 | 0 | 2016 | 16 |
| IL5 | Island | 17 | 0 | 0 | 2016 | 17 |
| IL6 | Island | 22 | 0 | 0 | 2016 | 22 |
| IL7 | Island | 31 | 0 | 0 | 2016 | 31 |
| IL8 | Island | 19 | 0 | 0 | 2015 | 19 |
| IL9 | Island | 20 | 0 | 0 | 2015/2016 | 20 |
| IL10 | Island | 0 | 0 | 24 | 2014 | 24 |
| IL11 | Island | 17 | 0 | 0 | 2015 | 17 |
| IL12 | Island | 24 | 0 | 0 | 2015 | 24 |
| IL13 | Island | 48 | 0 | 0 | 2015 | 48 |
| IL14 | Island | 32 | 0 | 0 | 2015 | 32 |
| IL15 | Island | 23 | 0 | 5 | 2016 | 28 |
| IL16 | Island | 15 | 0 | 0 | 2015/2016 | 15 |
| IL17 | Island | 25 | 0 | 0 | 2013 | 25 |
| M1 | Mainland | 0 | 0 | 34 | 2012 | 31 |
| M2 | Mainland | 0 | 9 | 7 | 2016 | 16 |
| M3 | Mainland | 0 | 0 | 20 | 2012 | 20 |
| M4 | Mainland | 26 | 0 | 0 | 2016 | 26 |
| M5 | Mainland | 6 | 0 | 26 | 2016 | 32 |
